# A Pilot Study on Machining Difficult-to-Cut Materials with the Use of Tools Fabricated by SLS Technology

**DOI:** 10.3390/ma14185306

**Published:** 2021-09-14

**Authors:** Mariusz Deja, Dawid Zieliński

**Affiliations:** Department of Manufacturing and Production Engineering, Institute of Machine and Materials Technology, Faculty of Mechanical Engineering and Ship Technology, Gdańsk University of Technology, G. Narutowicza Str. 11/12, 80-233 Gdańsk, Poland; dawid.zielinski@pg.edu.pl

**Keywords:** difficult-to-cut materials, finishing processes, rapid tooling, additive manufacturing, diamond grains, abrasive machining

## Abstract

The growing use of contemporary materials in various industrial sectors, such as aerospace, automotive, as well as the oil and gas industry, requires appropriate machining methods and tools. Currently, apart from the necessity to obtain high-dimensional and shape accuracy, the efficiency and economic aspects of the selected manufacturing process are equally important, especially when difficult-to-cut materials, such as hard and brittle ceramics, have to be machined. In the research presented in this paper, a prototype tool fabricated from polyamide powder by the SLS method was used in flat-lapping of Al_2_O_3_ ceramics, showing the promising potential and efficacy of rapid tooling and manufacturing in the area of abrasive machining. The influence of the selected input process factors, such as machining time, the type of abrasive suspension, kinematic parameters, and unit pressure, on technological effects, was analyzed. The microscopic observations of the active surface of the prototype tool showed its reinforcement with loose diamond abrasive particles (size D107), resulting in the effective material removal and improved surface finish of Al_2_O_3_ ceramic samples. The directions for further development of tools fabricated by the SLS method for applications in abrasive machining were also envisaged by the authors.

## 1. Introduction

Processing difficult-to-cut materials, such as superalloys, composites, titanium alloys, or advanced ceramics, contributes to the continuous development of finishing methods and abrasive tools. Conventional abrasive processes, such as grinding, lapping, polishing, and superfinishing, are still commonly used for finishing mechanical parts. Simultaneously, the production of components with increasingly complex geometries, e.g., by additive technologies, and the growing requirements from the industrial sectors make it necessary to develop new processes and tools, such as grinding with lapping kinematics using wheels with diamond or cubic boron nitride grains [1,2,3,4]. A study of the use of electroplated diamond wheels in lap-grinding, which allowed for effective machining of ceramic materials, was performed in [5,6,7,8]. Other examples of modern finishing processes based on abrasion are chemical mechanical polishing (CMP) [9], electrochemical polishing (ECP) [10], or ultrasonic cavitation abrasive finishing (UCAF) [11]. 

Ceramics, due to their superior properties such as good strength properties, low density, high hardness, as well as high temperature and chemical resistance, are widely used in many industrial areas and modern technological processes [12]. Ceramic cutting tools can be used for high-speed milling of heat-resistant superalloy IN718 [13]. Technical oxide ceramics Al_2_O_3_ is commonly used for making, among others, electronic and electrical components [14,15]. On the other hand, hard and brittle ceramics are very difficult to machine. Machining ceramic materials is still a big challenge for the industry [16]. Deja et al. pointed out [17] that fabricating abrasive tools by additive manufacturing (AM) processes is one of the promising directions in the development of novel finishing processes. This is due to the fact that AM technologies offer new opportunities in the fabrication of parts with strictly defined inner and outer geometries [18,19,20]. Moreover, even for abrasive tools with relatively simple geometric shapes, as in the case of lapping plates, AM technologies are cost-effective solutions for unit and small batch production, dedicated mainly for the fabrication of prototype tools. Less equipment is needed for production and postprocessing compared to subtractive technologies [16]. Powder bed fusion 3D-printing technologies based on metal alloys are widely used in the production of grinding wheels with a porous structure [21,22,23,24]. AM-based methods using curable resins can be used in the fabrication of lapping or polishing plates. Resin lapping wheels containing diamond abrasive grains enabled performing effective machining of hard and brittle ceramic materials [25,26]. Tools fabricated by selective laser sintering (SLS) can find application in grinding [27] or in lapping.

The SLS method used to produce lapping tools has rarely been studied, as indicated by Deja and Zieliński [17]. Herein, a prototype lapping plate was prepared by the SLS method and applied in the flat-lapping of technical ceramics Al_2_O_3_. The fabricated tool enabled the efficient material removal from hard and brittle ceramic samples. In the experimental tests, the influence of particular input process factors on the linear material loss and on the selected parameters of the geometrical structure of the machined surfaces was analyzed. As a result of the performed analysis, a determined set of machining parameters allowed obtaining technological effects characterized by good surface finish and relatively high material removal. 

## 2. Materials and Methods

**Figure 1:**
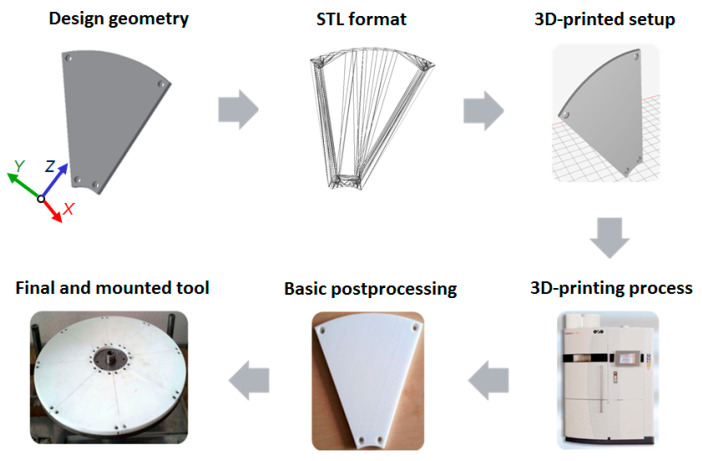
The 3D printing process chain of the SLS-lapping plate.

**Figure 2:**
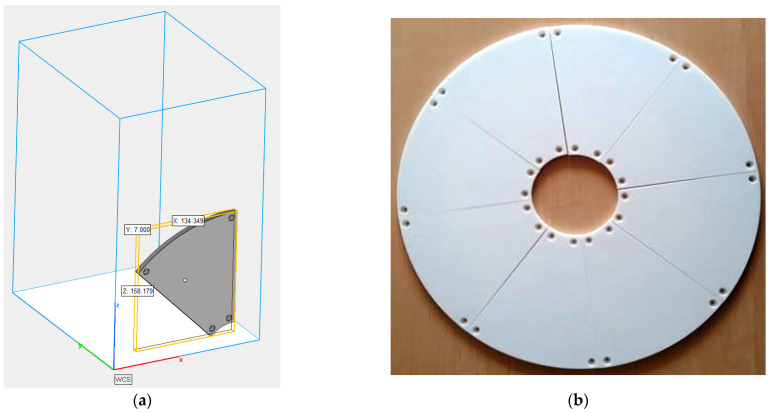
SLS-printed tool: (a) view of the printing position and dimensions of a single abrasive segment; (b) a prototype lapping plate consisting of eight abrasive segments.

The prototype abrasive tool was made of polyamide PA 2200 powder by the selective laser sintering (SLS) method using an industrial EOS Formiga P100 3D printer (EOS GmbH, Krailling, Germany) according to the process chain given in Figure 1 and the set of process parameters presented in Table 1. Due to the limited space of the 3D printer’s working chamber, the tool was divided into identical segments, which were then screwed to the metal body. The prototype lapping plate was composed of eight abrasive segments forming the uniform flat active surface of the tool (Figure 2). The cost of the fabrication of the prototype tool was approximately USD 200. 

Experimental tests of the lapping samples from Al_2_O_3_ were carried out on a machine tool (Gdańsk University of Technology, Gdańsk, Poland) equipped with two independent and programmable drive units of the lapping plate and leading ring [6]. The machine configuration allowed for the analysis of various kinematic parameters of the lapping process, which were changed using programmable indexers of stepper motors. Three identical cylindrical samples made of technical ceramics Al_2_O_3_ (HV10 = 1100 MPa) with an outer diameter d_w_ = 34 mm and an initial height h_w0_ = 30 mm were used in the experimental tests. The diamond paste with grain size SD 28/20 and volumetric grain concentration in abrasive paste 20%, as well as loose diamond grains D107 with machining oil were used as the abrasive suspension (*a_s_*). After a single test, the height of the samples was measured using a Mitutoyo micrometer (Mitutoyo, Kawasaki, Japan) with a resolution of 0.001 µm. Additionally, the surface roughness and waviness measurements were conducted using a contact profiler HOMMEL TESTER T500 according to the DIN4777 standards. A total of nine measurements of each type were made on each sample. In addition, the Sneox 3D optical profiler (Sensofar, Barcelona, Spain) was used to perform three-dimensional measurements of the surface topography of the Al_2_O_3_ samples.

## 3. Experimental Results

### 3.1. Scheme of Experimental Tests and Process Factors

**Figure 3:**
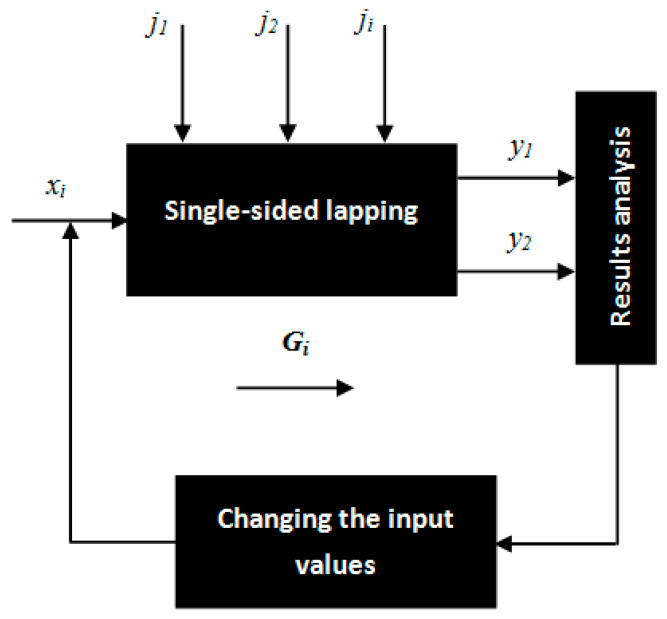
Scheme of the research tests of single-sided lapping with the prototype tool made by the SLS method. Characteristics of factors: X—independent variables: *x*_1_—type of abrasive suspension *a_s_*: diamond paste with SD 28/20 diamond grains; D107 diamond grains with oil; *x*_2_—unit pressure *p*: 6 kPa; 12 kPa; *x*_3_—lapping time *t*: 60 s, 120 s, 240 s, 300 s, 480 s, 720 s; 940 s; *x*_4_—rotational speed of the tool *n_t_*: 60 rev/min, 120 rev/min; *x*_5_—rotational speed of the leading ring (separator with workpieces) *n_w_*: 31 rev/min; 60 rev/min. Y—dependent variables: *y*_1_—linear material removal ∆*h*; *y*_2_—surface roughness and waviness: Ra, Wa. G—constant factors during all tests: *g*_5_—the machine tool for flat-lapping processes; *g*_6_—direction of the rotation of the lapping plate and leading ring; *g*_7_—the prototype SLS-printed lapping plate consisting of independent abrasive segments and made of polyamide powder with constant 3D printing process parameters; *g*_8_—machining method: single-sided lapping of flat surfaces; *g*_9_—material and geometry of the machined samples: cylindrical samples made of Al_2_O_3_; *g*_10_—methods of measuring the output variables. J—disturbing factors: *j*_1_—inaccuracies in setting the kinematic parameters of lapping, quantity, and the method of spreading the abrasive paste on the surface of the lapping plate, *j*_2_—the variation of the physical-mechanical properties of the lapping plate and the machined workpieces.

The influence of the abrasive suspension *a_s_*, kinematic parameters related to the rotational speed of a lapping plate *n_t_* and a leading ring *n_w_*, machining time *t*, as well as unit pressure *p* on the technological effects were investigated during the experiments. The main purpose of this pilot study was to check the potential of the abrasive ability of the prototype tool demonstrated for the first time in the literature. Figure 3 shows the scheme of the experimental tests, taking into account the influence of particular input variables, constant values, and disturbing quantities on the output variables during single-sided lapping of Al_2_O_3_ ceramic samples.

### 3.2. Experimental Tests of Single-Sided Lapping of Al_2_O_3_ Ceramics

The set of process conditions and independent variables adopted during the experimental tests are given in Table 2. As this was the first usage of the tool fabricated by SLS technology in lapping, the values of particular process parameters given in Table 2 were selected for subsequent tests depending on the technological results obtained in the preceding tests.

**Figure 4:**
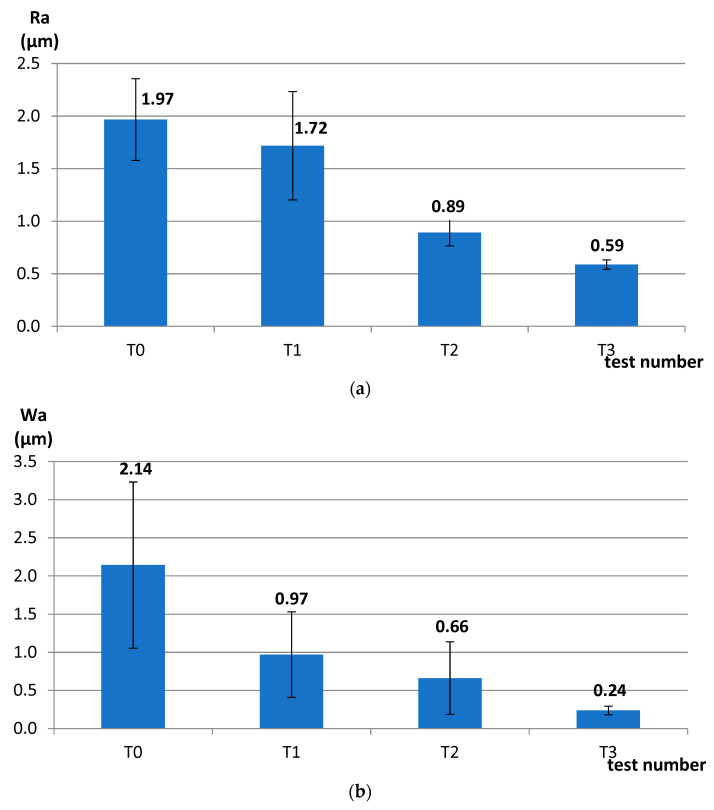
Average values of the roughness and waviness parameters after subsequent tests of single-sided lapping of Al_2_O_3_ technical ceramics using a prototype tool made by the SLS method (T0—initial state of samples before processing): (**a**) Ra parameter, (**b**) Wa parameter; error bars show ± the standard deviation.

In the first test T1, the use of relatively low process parameters, short machining time, and small diamond grains in the abrasive paste did not result in effective material cutting after 120 s (~1 µm), allowing only for a slight decrease in the roughness parameter Ra and a substantial decrease in the waviness parameter Wa (Figure 4). The obtained results indicated the need for an increase in the unit pressure *p* and kinematical parameters, according to Preston’s equation for the material removal rate, which can be written as:

(1) $$\frac{\Delta H}{\Delta t}=k\;\cdot \;p\;\cdot \;v$$

where *k* is the Preston constant, *p* is the nominal pressure, and *v* is the relative velocity. In addition, loose diamond grains D107 were added into the abrasive suspension to intensify the cutting process of hard ceramics in tests T2 and T3.

**Figure 5:**
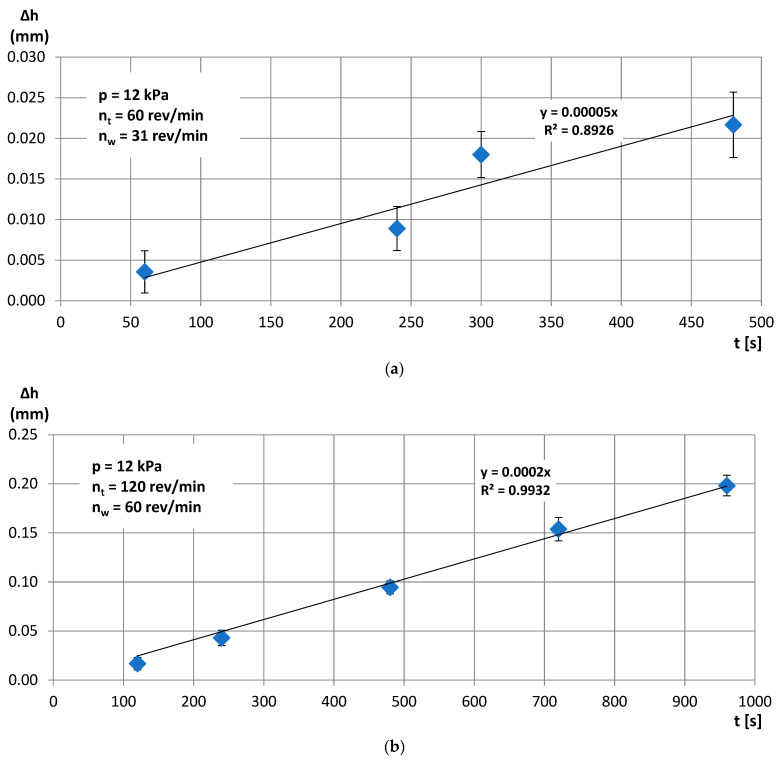
Cumulative linear material loss obtained in single-sided lapping of Al_2_O_3_ technical ceramics during tests T2 (**a**) and T3 (**b**); error bars show ± the standard deviation.

Increasing the pressure in test T2 to the value of *p* = 12 kPa allowed for a significant reduction in roughness and waviness parameters (Figure 4) and obtaining a satisfactory material loss (Figure 5a). In the last test T3, the values of the rotational speed of the lapping plate *n_t_* and the leading ring *n_w_* were doubled. This resulted in a significant improvement in the surface finish (Figure 4) and in high material removal, directly proportional to the machining time (Figure 5b). The material removal was four-times higher in test T3 after 480 s of machining with a better fitting of the experimental data to the trend lines. Thus, the slope of the equation of the trend line for test T3 was four-times higher than for test T2 with *R*^2^ = 0.99 and *R*^2^ = 0.89 for tests T3 and T2, respectively.

**Figure 6:**
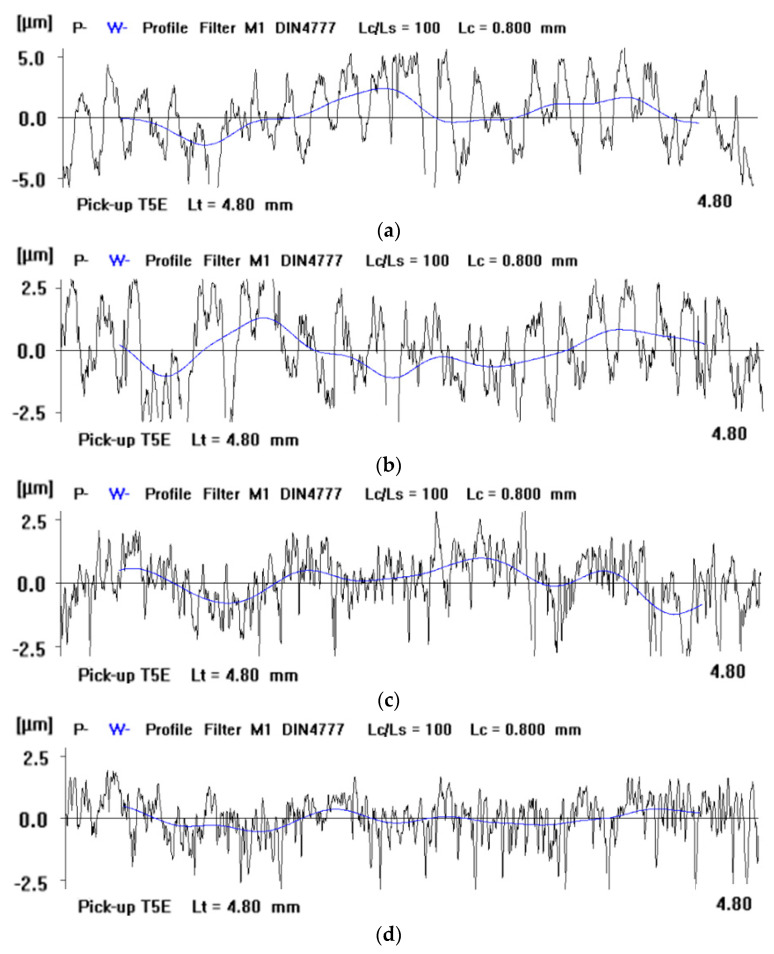
Comparison of the profiles measured on the surfaces of the Al_2_O_3_ ceramic samples: (**a**) initial state before machining, (**b**) after test T1, (**c**) after test T2, and (**d**) after test T3.

The selected profiles from the 2D measurements of the original and machined surfaces confirmed the significant improvement in the surface finish of the ceramic samples (Figure 6). Diamond grains embedded in the active surface of the soft tool allowed for effective cutting of the hard technical ceramics and improving the surface finish. The embedding of diamonds was confirmed during the microscopic observations of the tool’s active surface, as described in the following Section 3.3. 

### 3.3. Microscopic Observations of the Tool’s Active Surface

**Figure 7:**
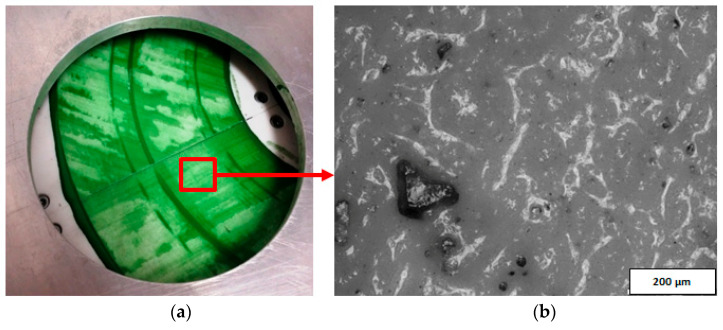
The active surface of the prototype tool with an abrasive suspension (**a**) containing diamond abrasive grits (**b**) after test T3.

**Figure 8:**
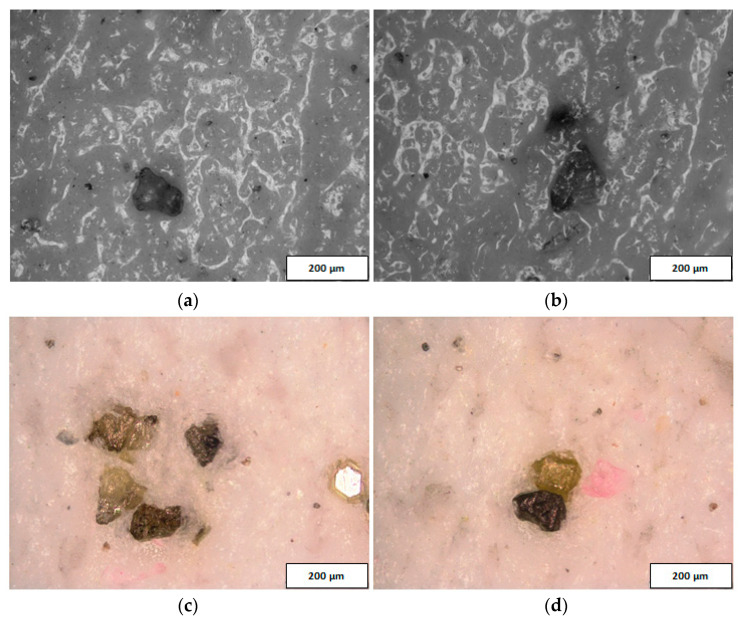
Selected sections of the tool’s active surface before (**a**,**b**) and after (**c**,**d**) cleaning from the abrasive suspension.

The active surface of the prototype abrasive tool was examined using a metallographic microscope OLYMPUS BX51 and dedicated OLYMPUS Stream Motion software. The analysis of the microscopic images confirmed the presence of diamond abrasive grits in the suspension located on the active surface of the tool, even after completing all tests (Figure 7). Diamond grits embedded in the active surface of the tool were also visible after its cleaning from the abrasive suspension. The grits presented in Figure 8 are of different sizes because the abrasive suspension contained mixed abrasive grits with small (D28) and large sizes (D107). Additionally, as a result of the interaction between the workpiece and the lap, the abrasive grits were crushed into smaller pieces. Grits and their fragments were distributed individually on the active surface of the prototype tool (Figure 8a,b), as well as in the form of characteristic conglomerates (Figure 8c,d).

**Figure 9:**
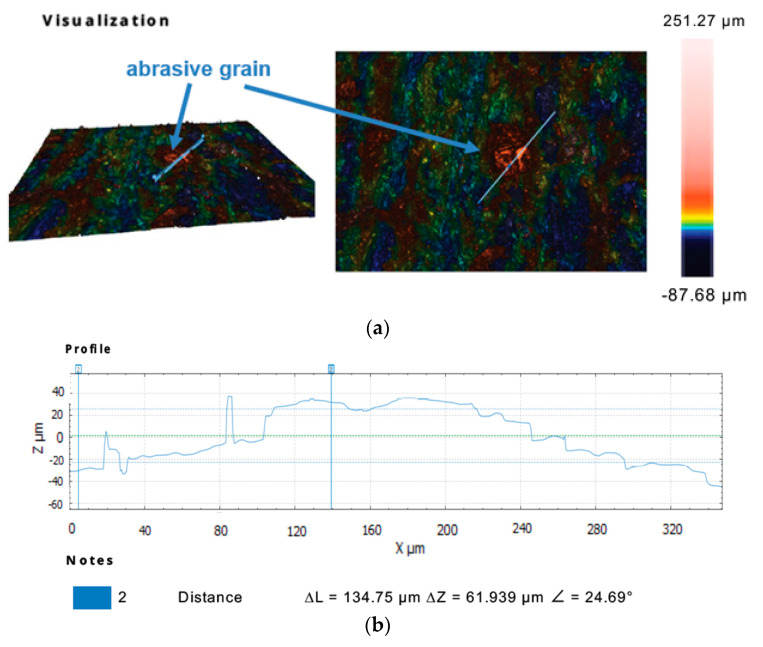
Profile extraction for a cross-section containing a diamond abrasive grain using confocal microscopy: a measurement area with the indicated place of an extracted profile (**a**) and the extracted profile (**b**).

**Figure 10:**
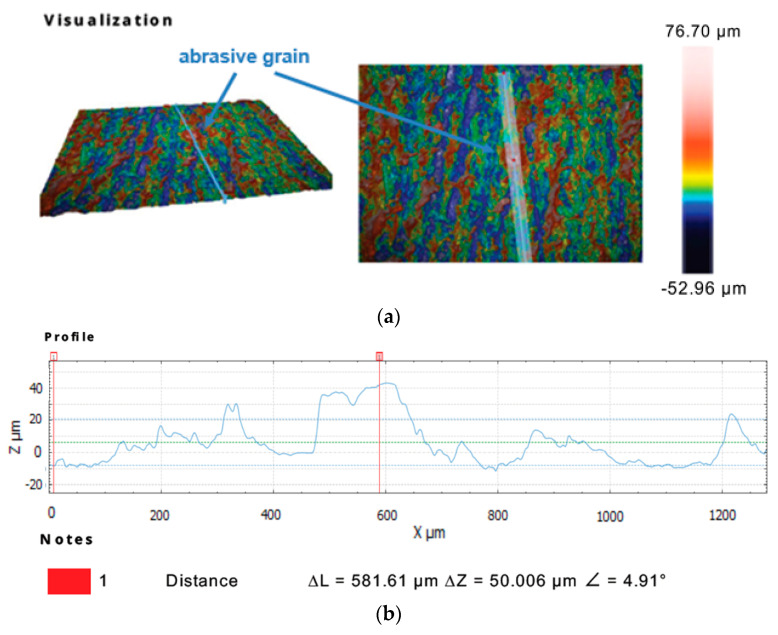
Profile extraction for a cross-section containing a diamond abrasive grain using interferometric microscopy: a measurement area with the indicated place of an extracted profile (**a**) and the extracted profile (**b**).

Further observations of the selected sections of the tool’s active surface with the use of the Sneox 3D optical profiler confirmed the exposure of diamond grits above the tool surface (Figure 9 and Figure 10).

### 3.4. Microscopic Observations of the Machined Surfaces of Al_2_O_3_ Technical Ceramics 

**Figure 11:**
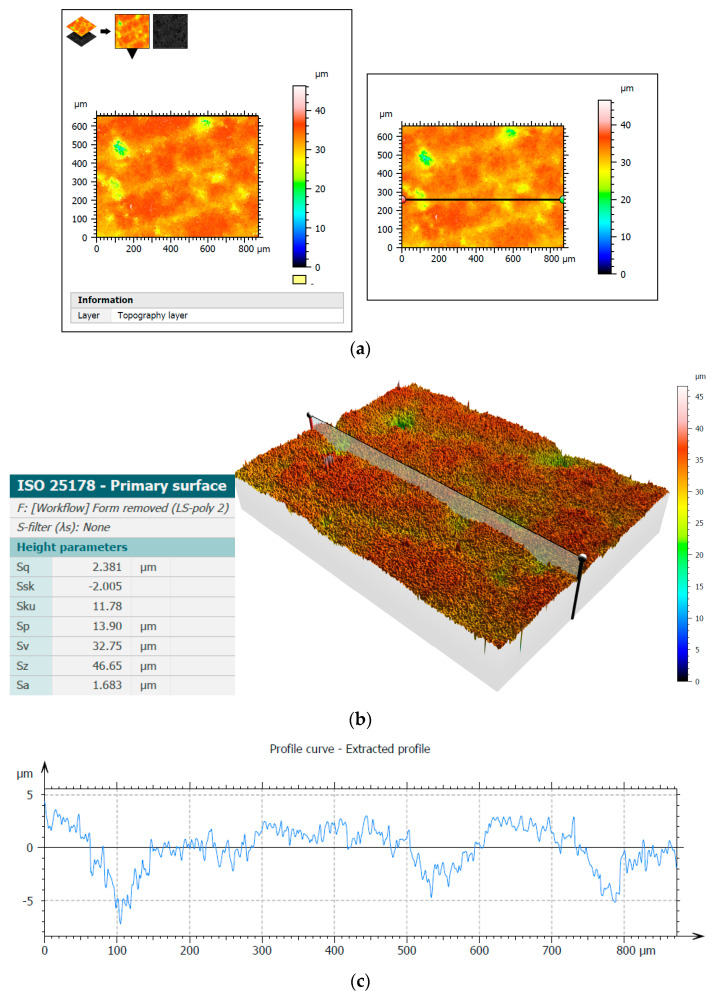
Three-dimensional measurement of the surface topography of the Al_2_O_3_ sample before experimental tests using confocal microscopy: measurement area with the indicated place of extracted profiles (**a**), surface topography with selected height parameters (**b**), and the extracted profile (**c**).

**Figure 12:**
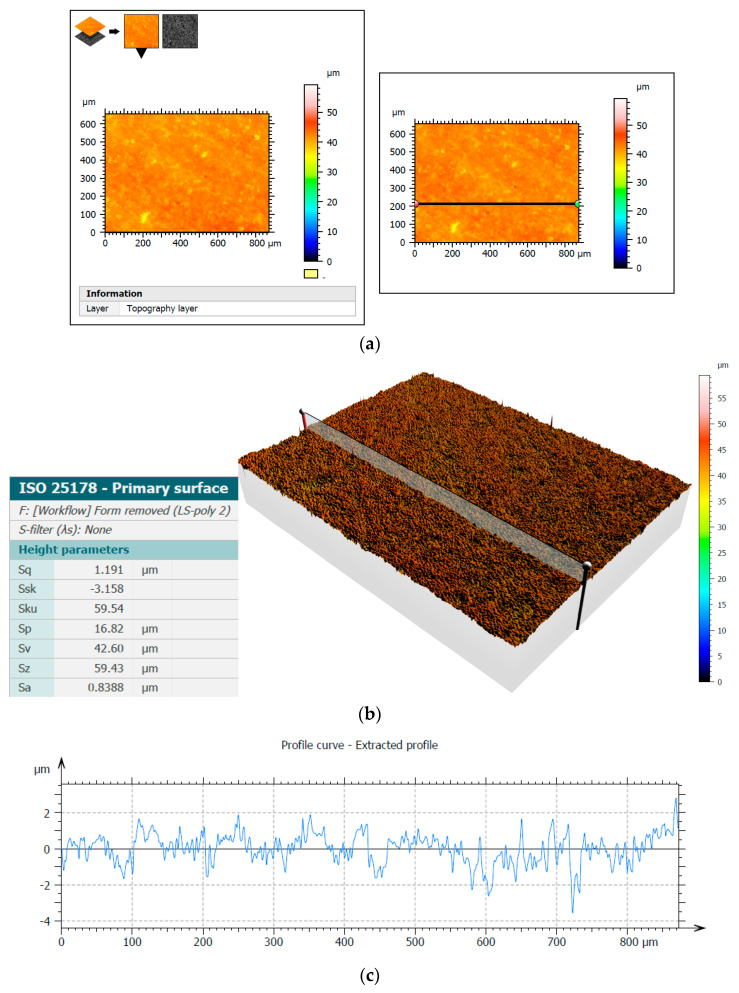
Three-dimensional measurement of surface topography of the Al_2_O_3_ sample after experimental tests using confocal microscopy: measurement area with the indicated place of extracted profiles (**a**), surface topography with selected height parameters (**b**), and the extracted profile (**c**).

The Sneox 3D optical profiler was used to examine the surface of Al_2_O_3_ samples. Figure 11 and Figure 12 show the surface topography before and after machining along with the extracted profiles with the selected height parameters. The primary surface was measured according to the ISO 25178 standards and roughness according to the ISO 4287 standards. 

The analysis of the microscopic images and profiles demonstrated a clear improvement in the surface quality, which was also confirmed by a noticeable decrease in the presented height parameters, e.g., the arithmetical mean height Sa decreased after test T3 from the initial value of 1.68 µm to the value of 0.84 µm. Moreover, the reduction in the Sq parameter value from 2.38 µm to 1.19 µm demonstrated surface smoothing, clearly visible on the three-dimensional surface topography (Figure 11b and Figure 12b). The values of the Ra parameter determined from the profiles presented in Figure 11c and Figure 12c were 1.59 µm and 0.60 µm, respectively, confirming a significant improvement in the surface finish and showing similar values to those obtained from the 2D measurements (Figure 4 and Figure 6).

## 4. Discussion

The analysis of the results obtained in the particular tests of the experimental study allowed the selection of the process parameters and the type of abrasive suspension for the effective performance of abrasive machining. The use of only SD 28/20 abrasive paste with D28 diamond grains allowed reducing the roughness and waviness of the machined surfaces, but with a very low material removal rate. Adding loose diamond grains of a bigger size D107 to the abrasive slurry resulted in a significantly higher removal of the ceramic material. Thus, the results of the experimental study confirmed that the use of larger abrasive grains enables effective machining of hard technical ceramics, while smaller grains can only be used to improve the surface quality of the machined parts for the applied tool made by the SLS method. The analysis of the spatial images and profiles obtained by confocal and interferometry techniques indicated the places where diamond abrasive grains were embedded. The applied values of the process parameters resulted in the embedding of diamond grains into the active surface of the tool, which was confirmed by microscopic observations (Figure 7 and Figure 8). The implementation of the profile extraction operation for the indicated cross-sections allowed for the estimation of the exposure of the selected grains, which was at the level of about 50 µm (Figure 9 and Figure 10). Thus, the soft material of the prototype tool and its porous structure resulting from the sintering of polyamide powder enabled the easy penetration of the diamond grains into the top layer of the tool’s active surface. Consequently, the relatively stable and permanent equipment of the prototype tool with diamond abrasive grains resulted in effective material removal (Figure 5) and a noticeable reduction in the surface roughness and waviness parameters of the Al_2_O_3_ ceramic samples (Figure 4 and Figure 6). This may suggest the transformation of the abrasion mechanism from a two-body abrasion characteristic for conventional lapping to a more effective three-body abrasion characteristic for grinding or lap-grinding.

## 5. Conclusions

The authors used the selective laser sintering (SLS) method for the production of prototype constructions of abrasive tools applied in flat-lapping technology. The optimistic results of the first experimental tests confirmed the great potential of using this type of tool in lapping technology and the advisability of conducting further research. The use of a prototype SLS-printed tool, with the change of particular input variables, made it possible to remove the material effectively from the Al_2_O_3_ samples and to improve their surface quality significantly. The obtained machining effects allowed the formulation of the following conclusions:

- The use of low kinematic parameters, unit pressure, as well as a short machining time and small abrasive grains did not allow for effective material removal and significant improvement in the surface quality of the samples made of Al_2_O_3_ technical ceramics, except for their waviness;
- An increase in the kinematic parameters, unit pressure, and machining time, as well as adding free large D107 abrasive grains significantly improved the amount of removed material, as well as the surface roughness and waviness parameters measured on the machined surfaces of the Al_2_O_3_ samples;
- A soft and resilient polyamide PA 2200 with flexural modulus 1500 MPa [28] induced the easy embedding of hard diamond grains. Microscopic observations conducted after the experimental tests confirmed the equipment of the tool’s active surface with diamond grains. Their permanent embedding in the lapping tool allowed for effective material removal along with the reduction in surface roughness and waviness. Microscopic observations and the obtained technological effects suggested the process transition from conventional lapping treated as three-body abrasion to grinding or lap-grinding treated as two-body abrasion;
- After all tests, no visible signs of damage or excessive wear of the prototype lapping plate were observed. After cleaning the remains of the applied abrasive suspension, the SLS-printed tool was effectively used in the next experimental tests.

In future research, the flat surface of the tool will be analyzed in detail, along with a discussion of the cutting mechanism characteristic for processing ceramics with the use of a lapping disc fabricated by SLS technology. Experiments will be carried out to determine the optimal process parameters and to design a tool with a structure allowing for better technological effects.

## Figures and Tables

**Table 1:** Specification of the lapping tool and the parameters of the SLS process.

**Lapping Plate**	
Outer tool diameter	d_o_ = 380 mm
Inner tool diameter	d_i_ = 90 mm
Raw material	polyamide powder PA 2200
**SLS Process Parameter**	
Thickness of a single layer	100 µm
Laser type	CO_2_
Power of laser	30 W
Length of laser beam	10.6 µm
Printing speed	10 mm/s
Printing resolution	0.005 mm
**Postprocessing**	
Sandblasting and cleaning of unsintered powder particles using compressed air	

**Table 2:** A set of machining parameters for pilot tests of single-sided lapping of technical ceramics Al_2_O_3._

X—Independent Variables					
Test Number	Unit Pressure *p* (kPa)	Rotational Speed (rev/min)		Type of Abrasive Suspension (mL)	Lapping Time Δ*t* (s)
		Lapping Tool *n_t_*	Leading Ring *n_w_*		
T1	6	60	31	4 mL diamond paste SD 28/20	60; 120
T2	12	60	31	0.5 mL of abrasive grains D107	60; 240; 300; 480
T3	12	120	60	0.5 mL of abrasive grains D107	120; 240; 480; 720; 940

## Data Availability

The data presented in this paper are available on request from authors.
